# Supplementary material for: The role of scientific communication in predicting science identity and research career intention
Source: PLoS One. 2020 Feb 19;15(2):e0228197. doi: 10.1371/journal.pone.0228197 (PMC7029843; doi:10.1371/journal.pone.0228197)
Supplement: Attachment — Submitted filename: Role scientific communication in predicting science identity and research career intentions.docx [file pone.0228197.s002.docx]

**Strengths**

- Much of the research and interventions that target STEM careers have focused on undergraduates with the goal of increasing the number of students, especially women and underrepresented minorities who pursue doctoral degrees. The erroneous assumption made here is that once individuals reach the doctoral or postdoctoral phase, pursuing a research career is the most likely or the most desired career pursuit. However, the data do not support this assumption as the proverbial “leaks” become more pronounced as individuals advance through the academic pipeline. Thus, the current study is an important contribution as it targets doctoral and postdoctoral trainees who are significantly underrepresented in STEM/biomedical research intervention studies.
- Additional areas of strength are the longitudinal design, sample diversity in terms of gender, race/ethnicity, and first generation status, and the inclusion of science specific constructs rather than focusing only on social identity constructs.
- Additional strength is the inclusion of scientific communication (and its operationalized components) within a broader model of intentions to pursue research career.
- The conceptual model presented in theoretically grounded and includes several well established and supported science intention constructs (e.g., science self efficacy, science identity) as well as social behavioral theories that show desired outcomes are more likely when expectancies and behavioral skills approximate intentions (e.g., SC skills, SC products, SC outcomes).

**Limitations**

- The study includes both doctoral trainees and postdoctoral students with the doctoral students outnumber postdocs 3 to 1. As one would expect, these are two very different groups especially in light of the study constructs—intentions to pursue research career and research products (presentations and publications). The authors do not provide data on the developmental stage of the doctoral students (i.e., average year in their doctoral program). Doctoral students in their first or second year will likely have fewer first author publications and conference presentations than more advanced students. It is unclear if the doctoral student sample represents a broad distribution in terms of years in program or are skewed either early or more advanced graduate students.
- The authors stated they recruited biomedical and behavioral sciences programs. Additional detail should be provided for how disciplines were included for recruitment especially in light of 25% of the sample falling into the “other” category. For instance, were clinical psychology programs included which provide training in both research and clinical practice versus behavioral science programs that focus only on research training?
- The authors posit a clear unidirectional framework that language skills precede and lead to identity—based primarily on social/cultural and cognitive theories of language development. However, there are some limitations to applying this framework to the current model of scientific communication and science identity. A science identity is one that is acquired and completely volitional which is not the case for many social identities (race, gender, cultural) where language precedes identity formation. Is it not possible that in the context of scientific career intentions, the relationship between scientific communication and science identity is bidirectional instead of unidirectional? Is it not possible for a science identity to develop before one has acquired scientific communication skills and that science identity facilitates (or motivates) one to acquire SC skills? Moreover, the current sample includes doctoral students and postdocs in biomedical research disciplines, which one could assume already have some degree of science identity prior to entering their doctoral programs, yet science identity is not measured at Time 1.
- This is a longitudinal dataset but it is unclear why some constructs were measured at multiple time points and others at single time points. For instance, SC productivity was measured only at Time 1 and SC outcome expectations at Time 3. Why would we not expect SC productivity to increase over time as participants advance through their training programs and that productivity may directly predict more proximal SC outcomes (Time 1 experiences predict Time 2 outcomes, etc). And what about length of time in program as a covariate for SC Productivity---productivity more likely for advanced doctoral students.
- The authors point to the dyadic relationship (mentor-trainee) as a novel contribution to the current study but only present the trainee data rather than dyadic analyses.
- There are a couple of discrepancies between the variables in Table 3 and the description in the methods section. In the text women are coded as 1 but as 0 in the table. Similarly, in the text first gen is coded as 1 but as 0 in the table.
- The authors also make a couple of conclusions in the discussion that did not appear to be reflected in their analyses. For instance in the following sentence, “Scientific writing, presenting, and spontaneous speaking (conversation) all contributed to these effects, and effects were not dependent on the perceived “quality” of the skills or success in publication. Rather, they stemmed from frequency of engagement.” It is not clear to me how the authors can discount “quality” when they did not report measuring quality but only measured frequency (and limited frequency with some measures due to item/scale limitations).
- The authors did address some of the scale limits in their discussion; however, this could be expanded a bit more. Each of the SC scales had items that did not load on derived factors and were subsequently omitted from analyses. The most problematic of which were SC self-efficacy with 40% of the items not loading and SC outcome expectations with 50% of the items not loading. Was there limited prior validity work using these scales or something about the current sample to explain the scale differences?
