# Supplementary material for: The role of scientific communication in predicting science identity and research career intention
Source: PLoS One. 2020 Feb 19;15(2):e0228197. doi: 10.1371/journal.pone.0228197 (PMC7029843; doi:10.1371/journal.pone.0228197)
Supplement: Attachment — Submitted filename: Response to Reviewers.docx [file pone.0228197.s003.docx]

**PONE-D-19-15861**	December 4, 2019

**“The role of scientific communication in predicting science identity and research career intention”**

**RESPONSE TO REVIEWERS**

We thank the reviewers for their thoughtful and constructive reviews. We recognize that the interdisciplinary nature of the study adds complexity to the review process and are grateful to the reviewers for the extra effort involved in evaluating it.

We are sending both a Track Changes version and a clean version of the document for the reviewers’ convenience in identifying changes. Some additional copy editing was done that was not requested by the reviewers. In addition, we noted that the Strengths and Limitations section was improperly placed and have moved it to just below the Discussion header.

Below we address reviewer comments. Please note that line numbers refer to the clean version of the document; relevant changes are highlighted in the Track Changes version of the document.

**Reviewer 1**

Summary and overall impression: The article makes a nice contribution to the understanding of career thinking among biomedical trainees by calling attention to scientific communication and its relationship to science identity. This is novel and useful research. It is well-written with narrative that corresponds nicely with the charts and figures. The integration of scientific communication constructs within SCCT will be helpful to anyone concerned with supporting and developing biomedical PhD students

Areas for improvement: I found the article well-written and clear, so my comments fall into the “minor issue” category. I hope that attention to these points will improve an already strong paper:

a. Because career intention is an important focus of this paper, I suggest some attention to clarifying and staying consistent with terminology. I see “research career” in the title, “academic faculty career” in the abstract, “research career intention” (p. 16), and “academic career intention” used in Figure 1. Do these all refer to a faculty career that is research-intensive, i.e., a PI? As I attempted to seek clarification, I reread the text section on p. 16 and then went to the table 3 – on table 3 I saw “intend to be a PI” and “intend to work in a higher ed academic setting” in table 3, which were not consistent with the text description on p. 16.

**We have corrected this ambiguity and changed the instances to ‘research career intention,’ LL. 31, 133-4, 153-4. Research career intention is a construct composed of “intend to be a PI” and “support science and research but not conduct research.”**

b. With science identity as a central focus, I wanted to know the items used to measure this construct with a bit more detail than provided. I feel the claims about science identity rest on this measurement of science identity thus necessitating the addition of details of what aspects of science identity were measured.

**We have added reference to Chemers’ science identity scale (2011) and provided an example of one of the items, LL. 320-2.**

c. In Table 2, first-gen college may need a definition (neither parent attained a bachelor’s degree?) as sometimes first-gen is defined differently. As well, I was curious what was used for the “economic standing” characteristic, although that variable was not mentioned in the text so it may not be necessary to include on the table.

**We have added footnotes providing definitions, LL. 267-70.**

d. When I saw the focus group quotation on p. 23, I did not recall seeing a mention of focus groups in the methodology section. Was this part of the current study or drawn from earlier research?

**We have clarified this reference to an earlier study, LL. 535-40.**

e. “Communities of Practice” may need citation (Lave & Wenger’s work, 1991) somewhere in the introductory/theoretical framing sections because this concept appears in the discussion section several times (p. 24, 30). Though an alternative is to reword without using the specific phrase, community of practice.

**Citation and definition added, LL. 528-30.**

f. “given that science identity is a significant predictor of career intentions” (p. 28/29) – may be too strong a statement as even some studies cited previously in the paper (Estrada, et al.) on p. 9 “did not predict career outcomes.”

**Statement was moderated, LL. 613-4.**

Other: Given that trainees often bristle at critical feedback and may feel singled out, I think another audience for this work is the trainees themselves. The idea of scientific communication as a distinct language variety that can improve through practice and frequency is powerful: it normalizes language development in a particular field as a process that everyone goes through to be recognized in that field, both by oneself and by others.

**Thank you. We agree.**

I am not an expert in statistics (which is why I answered "do not know" for the statistical analysis question in this review), but given that the statistics are accurate, I found it easy to follow the findings because of the good textual descriptions that corresponded with Table 3 and Figure 1.

**Reviewer 2**

**Strengths**

- Much of the research and interventions that target STEM careers have focused on undergraduates with the goal of increasing the number of students, especially women and underrepresented minorities who pursue doctoral degrees. The erroneous assumption made here is that once individuals reach the doctoral or postdoctoral phase, pursuing a research career is the most likely or the most desired career pursuit. However, the data do not support this assumption as the proverbial “leaks” become more pronounced as individuals advance through the academic pipeline. Thus, the current study is an important contribution as it targets doctoral and postdoctoral trainees who are significantly underrepresented in STEM/biomedical research intervention studies.
- Additional areas of strength are the longitudinal design, sample diversity in terms of gender, race/ethnicity, and first generation status, and the inclusion of science specific constructs rather than focusing only on social identity constructs.
- Additional strength is the inclusion of scientific communication (and its operationalized components) within a broader model of intentions to pursue research career.
- The conceptual model presented in theoretically grounded and includes several well established and supported science intention constructs (e.g., science self efficacy, science identity) as well as social behavioral theories that show desired outcomes are more likely when expectancies and behavioral skills approximate intentions (e.g., SC skills, SC products, SC outcomes).

**Limitations**

- The study includes both doctoral trainees and postdoctoral students with the doctoral students outnumber postdocs 3 to 1. As one would expect, these are two very different groups especially in light of the study constructs—intentions to pursue research career and research products (presentations and publications). The authors do not provide data on the developmental stage of the doctoral students (i.e., average year in their doctoral program). Doctoral students in their first or second year will likely have fewer first author publications and conference presentations than more advanced students. It is unclear if the doctoral student sample represents a broad distribution in terms of years in program or are skewed either early or more advanced graduate students.

**Participants were not asked their year in their doctoral or postdoctoral program. However, they were asked how many years they had worked with their primary mentor. At the Time 1 survey, 14% of doctoral trainees had worked with their current primary mentor less than one year. Thus, we assumed that the number of doctoral students in the early years of their program was small.**

**As you mention, we did assume that senior trainees had higher productivity than junior ones. However, even though senior doctoral or postdoctoral trainees had more SciComm productivity than earlier-stage doctoral trainees, we assumed that the construct relationships between *productivity and self-efficacy* in our model would have similar patterns regardless of their years enrolled in the program or academic rank. That is, trainees who had higher productivity have higher self-efficacy, regardless of their demographic characteristics. Moreover, in order to examine whether academic rank or years enrolled in the program moderate the relations specified in the model, we would need to estimate the same structural model in each of two or more different groups (e.g., doctoral vs. postdoctoral) and then compare the structural relationships across the groups. Unfortunately, we did not have the sample size necessary to perform such multi-group analyses. Future studies are needed using multi-group structural equation modeling to see if the relationships among the constructs differ by gender, rank, race/ethnicity.**

- The authors stated they recruited biomedical and behavioral sciences programs. Additional detail should be provided for how disciplines were included for recruitment especially in light of 25% of the sample falling into the “other” category. For instance, were clinical psychology programs included which provide training in both research and clinical practice versus behavioral science programs that focus only on research training?

**We have broken out the ‘other’ category and provided additional information in Table 2 (LL. 258-9, 265-6), as well as explaining the exclusion criterion that only trainees *with research as their primary focus* were eligible (LL. 233-4). Many in the ‘other’ category had reported other STEM research disciplines, such as Chemistry.**

- The authors posit a clear unidirectional framework that language skills precede and lead to identity—based primarily on social/cultural and cognitive theories of language development. However, there are some limitations to applying this framework to the current model of scientific communication and science identity. A science identity is one that is acquired and completely volitional which is not the case for many social identities (race, gender, cultural) where language precedes identity formation. Is it not possible that in the context of scientific career intentions, the relationship between scientific communication and science identity is bidirectional instead of unidirectional? Is it not possible for a science identity to develop before one has acquired scientific communication skills and that science identity facilitates (or motivates) one to acquire SC skills? Moreover, the current sample includes doctoral students and postdocs in biomedical research disciplines, which one could assume already have some degree of science identity prior to entering their doctoral programs, yet science identity is not measured at Time 1.

**This comment touches on a number of important theoretical and philosophical questions. As a brief response, we agree that a bidirectional relationship between identity and language use is very likely. We have included a statement speculating on the possibility of a feedback loop between science identity and SC in LL. 546-50, where the relationality principle is mentioned. Because we were testing an SCCT model, which posits performance accomplishments (operationalized as SC productivity) as an exogenous variable preceding identity, reciprocal effects were not measured. We have added a statement regarding this point in LL. 497-500.**

**Regarding the possibility of having science identity before acquiring SC skills, our data do show, congruent with sociolinguistic perspectives, that trainees with higher productivity in SC at Time 1 have higher science identity at Time 2, through SC self-efficacy. On a more abstract level, we suspect that while trainees in earlier stages may have a strong *interest* in and intention to pursue research, trainees in these later, more committed stages may not endorse the identity of ‘bona fide’ scientist until they are somewhat fluent in communicating their research. (This is reflected in comments that have been made to us not only by trainees, but by senior faculty who say they themselves didn’t feel like they were “*really* scientists” until they participated in a study section and were able to converse at the level of the other members.)**

**Whether any identity, professional or social/ethnic, is either completely volitional or non-volitional is an open question, especially with respect to language use. Language use is partly conscious and deliberate and partly unconscious and automatic, which is part of what makes it so interesting. What we feel is important is that mentors recognize the power of acting on communication skills early in training rather than waiting until a high-stakes product is at hand.**

- This is a longitudinal dataset but it is unclear why some constructs were measured at multiple time points and others at single time points. For instance, SC productivity was measured only at Time 1 and SC outcome expectations at Time 3. Why would we not expect SC productivity to increase over time as participants advance through their training programs and that productivity may directly predict more proximal SC outcomes (Time 1 experiences predict Time 2 outcomes, etc). And what about length of time in program as a covariate for SC Productivity---productivity more likely for advanced doctoral students.

**We did not measure all constructs at every time point because construct placements were based on our theoretical model. Social-cognitive career theory posits that previous experiences (SC productivity) predict self-efficacy (SC self-efficacy). We did assume that SC productivity increased over time, but we did not measure SC productivity at every time point (6-7 months interval).**

- The authors point to the dyadic relationship (mentor-trainee) as a novel contribution to the current study but only present the trainee data rather than dyadic analyses.

**We have removed the reference to the novelty of the dyadic nature of the study (LL. 470).**

- There are a couple of discrepancies between the variables in Table 3 and the description in the methods section. In the text women are coded as 1 but as 0 in the table. Similarly, in the text first gen is coded as 1 but as 0 in the table.

**Corrected, LL. 377-8.**

- The authors also make a couple of conclusions in the discussion that did not appear to be reflected in their analyses. For instance in the following sentence, “Scientific writing, presenting, and spontaneous speaking (conversation) all contributed to these effects, and effects were not dependent on the perceived “quality” of the skills or success in publication. Rather, they stemmed from frequency of engagement.” It is not clear to me how the authors can discount “quality” when they did not report measuring quality but only measured frequency (and limited frequency with some measures due to item/scale limitations).

**We have added more nuance to the reference to ‘quality’. LL. 460-6.**

- The authors did address some of the scale limits in their discussion; however, this could be expanded a bit more. Each of the SC scales had items that did not load on derived factors and were subsequently omitted from analyses. The most problematic of which were SC self-efficacy with 40% of the items not loading and SC outcome expectations with 50% of the items not loading. Was there limited prior validity work using these scales or something about the current sample to explain the scale differences?

**Some of these results on the CFA item loadings do stem from current sample. First, sample sizes of two studies were different; n=182 for the current study, but n= 511 for the previous study. Second, sample characteristics were different; the current sample was 71% doctoral and 29% postdoctoral; the previous sample was 48% doctoral and 50% postdoctoral. Third, we used a more stringent SEM fit criteria for the current study; e.g., self-efficacy: RMSEA=.055 for the current study, but RMSEA =.091 for the previous study.**

**In addition, the 50% figure given for low item loadings on outcome expectations is misleading. Actually, only one item from the original outcome expectations measure was dropped. We wrote five new, additional items for this variable to test in the current sample. None of them were statistically satisfactory, so we dropped all five. In retrospect, it is needless to state this in the paper, so this has been removed from the Measures section and the section has been rewritten. See LL. 310-8.**

**Journal Requirements:**

1. When submitting your revision, we need you to address these additional requirements. Please ensure that your manuscript meets PLOS ONE's style requirements, including those for file naming. The PLOS ONE style templates can be found at <http://www.journals.plos.org/plosone/s/file?id=wjVg/PLOSOne_formatting_sample_main_body.pdf> and <http://www.journals.plos.org/plosone/s/file?id=ba62/PLOSOne_formatting_sample_title_authors_affiliations.pdf>

2.  Please provide additional details regarding participant consent. In the ethics statement in the Methods and online submission information, please ensure that you have specified whether consent was informed. If the need for informed consent was waived by the ethics committee, please include this information.

**Corrected, LL. 239.**

3.  We note that you have indicated that data from this study are available upon request. PLOS only allows data to be available upon request if there are legal or ethical restrictions on sharing data publicly. For more information on unacceptable data access restrictions, please see <http://journals.plos.org/plosone/s/data-availability#loc-unacceptable-data-access-restrictions>.

In your revised cover letter, please address the following prompts:

a) If there are ethical or legal restrictions on sharing a de-identified data set, please explain them in detail (e.g., data contain potentially sensitive information, data are owned by a third-party organization, etc.) and who has imposed them (e.g., an ethics committee). Please also provide contact information for a data access committee, ethics committee, or other institutional body to which data requests may be sent.

b) If there are no restrictions, please upload the minimal anonymized data set necessary to replicate your study findings as either Supporting Information files or to a stable, public repository and provide us with the relevant URLs, DOIs, or accession numbers. For a list of acceptable repositories, please see <http://journals.plos.org/plosone/s/data-availability#loc-recommended-repositories>.

**We are including our data file as Supporting Information.**

We will update your Data Availability statement on your behalf to reflect the information you provide.
